# Statistical Modeling Suggests that Antiandrogens in Effluents from Wastewater Treatment Works Contribute to Widespread Sexual Disruption in Fish Living in English Rivers

**DOI:** 10.1289/ehp.0800197

**Published:** 2009-01-07

**Authors:** Susan Jobling, Robert. W. Burn, Karen Thorpe, Richard Williams, Charles Tyler

**Affiliations:** 1 Institute for the Environment, Brunel University, Uxbridge, Middlesex, United Kingdom;; 2 Beyond the Basics Ltd., Burnham, Buckinghamshire, United Kingdom;; 3 Statistical Services Centre, School of Biological Sciences, University of Reading, Reading, United Kingdom;; 4 Ecotoxicology and Aquatic Biology Research Group, School of Biosciences, University of Exeter, Exeter, Devon, United Kingdom;; 5 CEH Wallingford (Centre for Ecology & Hydrology), Wallingford, Oxfordshire, United Kingdom

**Keywords:** antiandrogen, endocrine disruption, estrogen, feminization, fish, testicular dysgenesis

## Abstract

**Background:**

The widespread occurrence of feminized male fish downstream of some wastewater treatment works has led to substantial interest from ecologists and public health professionals. This concern stems from the view that the effects observed have a parallel in humans, and that both phenomena are caused by exposure to mixtures of contaminants that interfere with reproductive development. The evidence for a “wildlife–human connection” is, however, weak: Testicular dysgenesis syndrome, seen in human males, is most easily reproduced in rodent models by exposure to mixtures of antiandrogenic chemicals. In contrast, the accepted explanation for feminization of wild male fish is that it results mainly from exposure to steroidal estrogens originating primarily from human excretion.

**Objectives:**

We sought to further explore the hypothesis that endocrine disruption in fish is multicausal, resulting from exposure to mixtures of chemicals with both estrogenic and antiandrogenic properties.

**Methods:**

We used hierarchical generalized linear and generalized additive statistical modeling to explore the associations between modeled concentrations and activities of estrogenic and antiandrogenic chemicals in 30 U.K. rivers and feminized responses seen in wild fish living in these rivers.

**Results:**

In addition to the estrogenic substances, antiandrogenic activity was prevalent in almost all treated sewage effluents tested. Further, the results of the modeling demonstrated that feminizing effects in wild fish could be best modeled as a function of their predicted exposure to both antiandrogens and estrogens or to antiandrogens alone.

**Conclusion:**

The results provide a strong argument for a multicausal etiology of widespread feminization of wild fish in U.K. rivers involving contributions from both steroidal estrogens and xenoestrogens and from other (as yet unknown) contaminants with antiandrogenic properties. These results may add further credence to the hypothesis that endocrine-disrupting effects seen in wild fish and in humans are caused by similar combinations of endocrine-disrupting chemical cocktails.

Wildlife populations associated with the aquatic environment can be exposed to concentrations of endocrine-disrupting pollutants that are high enough to compromise their reproductive capacity (reviewed by Vos et al. 2000); this exposure may, in turn, have population-level consequences (Kidd et al. 2007). The widespread nature of these abnormalities has led to substantial interest from scientists and the general public. This concern stems, in part, from the hypothesis that reproductive diseases seen in humans are also caused by exposure to the same chemical contaminants (Skakkebaek et al. 2001). However, the actual evidence to support the wildlife–human connection is weak. Moreover, in most cases there is little evidence to link cause and effect in even a single species, let alone multiple species. Some of the best evidence has been found in riverine fish populations where feminization of wild male fish (e.g., Jobling et al. 1998) is thought to be caused predominantly by exposure to steroidal estrogens in wastewater treatment work (WWTW) effluents originating from human and animal excretion (Desbrow et al. 1998; Routledge et al. 1998), with minor contributions from other estrogenic chemicals found in WWTWs effluents, such as bisphenols and phthalates, nonylphenols (NPs) and their ethoxylates, and carboxylates (Gibson et al. 2005; Harries et al. 1997; Vajda et al. 2008; Vethaak et al. 2005).

Supporting the role of these steroidal estrogens in the feminization of wild fish, recently, a very strong correlation was shown between the predicted steroidal estrogen content of U.K. rivers and feminization in wild fish (Jobling et al. 2006). Reproductive disorders also seen in human males are, however, best induced by exposing laboratory rodents to environmentally relevant concentrations of antiandrogens and estrogens rather than to estrogens alone (Sharpe and Skakkebaek 2008; Skakkebaek et al. 2001), thus suggesting that the etiology of endocrine-disruptor–induced reproductive diseases likely differ in humans and fish. Notwithstanding this, the fact that there are > 100,000 substances in wastewater effluents (not including the different isomers of chemicals or their products of degradation), many of which have endocrine-disrupting properties other than estrogenic, makes it highly likely that the feminizing responses seen in male fish also have a multicausal etiology involving chemicals with nonestrogenic mechanisms of action. The objective of the present study, therefore, was to further explore this possibility by challenging the hypothesis that steroidal estrogens are solely responsible for widespread sexual disruption seen in wild fish in U.K. rivers. We used data on hormonal (estrogenic, antiestrogenic, androgenic, and antiandrogenic) activities and concentrations of known endocrine disruptors in WWTW effluents, together with hydrologic data, to predict hormone and antihormone concentrations in receiving waters over a wide geographic range. We then explored their relationships with sexual disruption in the wild fish living in these waters using statistical modeling. The results suggest that antiandrogenic chemicals of unknown identities are widespread in U.K. effluents and receiving waters and that, in addition to the steroidal estrogens, these constituents of WWTW effluents are likely to play a major role in causing endocrine disruption in wild fish.

## Methods

### Data sources

#### Effluent hormonal activity and chemistry

**Figure 1:**
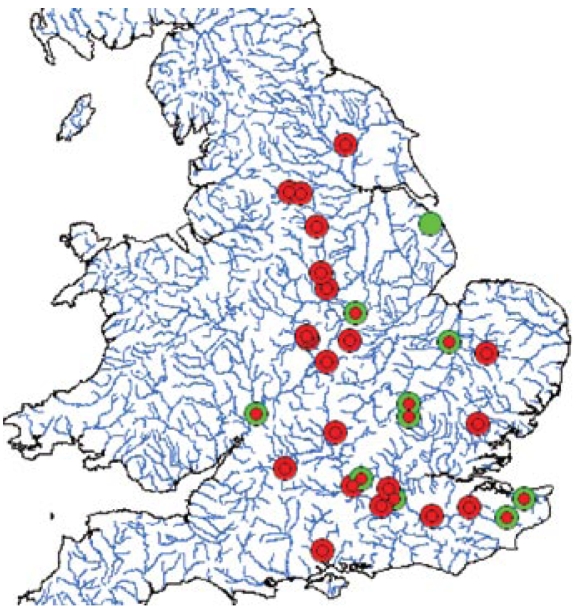
Map showing the overlap in spatial distribution of estrogenic (small circles) and antiandrogenic (large circles) activity in the U.K WWTWs sampled. Red indicates the presence of activity; green indicates that no activity was found.

The Environment Agency’s survey of hormonal activity in 51 effluents (Environment Agency 2007) provided data on effluent chemistry from the results of a U.K. national survey of sewage treatment works effluents (locations shown in Figure 1). In that study, samples were analyzed for 17β-estradiol (E_2_), estrone (E_1_), 17α-ethinylestradiol (EE_2_), 4-*tert*-nonyl phenol (NP), and lower NP ethoxylates (NPnEO, where *n* = 1–5 and indicates ethoxylate chain length) and for total estrogenic, antiestrogenic, androgenic, and antiandrogenic activity in recombinant yeast screens [rYES for (anti-)estrogenic and rYAS for (anti-)androgenic activities]. The rYES and rYAS were supplied by J. Sumpter (Brunel University), and the assays were run as described by Routledge and Sumpter (1996) and Sohoni and Sumpter (1998). The detailed methods for the chemical analysis have been fully described by the Environment Agency (2007) and in other articles in which these data have also been examined (Johnson et al. 2007; Thorpe et al. 2006). Steroid estrogens were detected in all effluents at concentrations consistent with previous observations, the relative persistence of the three steroidal estrogens and differences in human excretion rates.

#### Estimations of (anti-)androgenic and (anti-)estrogenic activity or steroidal estrogen and alkylphenol concentrations in the river water at the fish capture sites

In the present study, we identified 30 sites where modeled predictions of exposure to steroidal estrogens, NPs, and hormonal activities in the receiving environment could be made and where fish were also captured. The sites covered a wide geographical range and had a wide variation in the proportion of the flow of the river composed of sewage effluent.

For each site, we divided the concentrations of various estrogenic chemicals and hormonal activities in the effluents of WWTWs located upstream by the dilution factor in the river at the point of fish capture to obtain estimated concentrations of each parameter in the river. Methods and supporting references have been published previously (Jobling et al. 2006).

#### Measurements of sexual disruption in fish

We analyzed data from the Environment Agency’s spatial survey of sexual disruption in fish (Jobling et al. 2006), which provided data on the location and prevalence of male fish with elevated plasma vitellogenin (VTG) levels, a feminized reproductive duct (fem.duct) or with developing eggs (oocytes) in the testes, and on the severity of this condition [mean relative number of oocytes in the testes (fem.index); Nolan et al. 2001] in “male” roach from each of the 30 sites (Table 1). There were 1,083 fish in total (12–71 from each location). Feminized male fish (fish with feminized ducts and/or feminized germ cells) were present at many of these sites.

### Statistical methods

**Figure 2:**
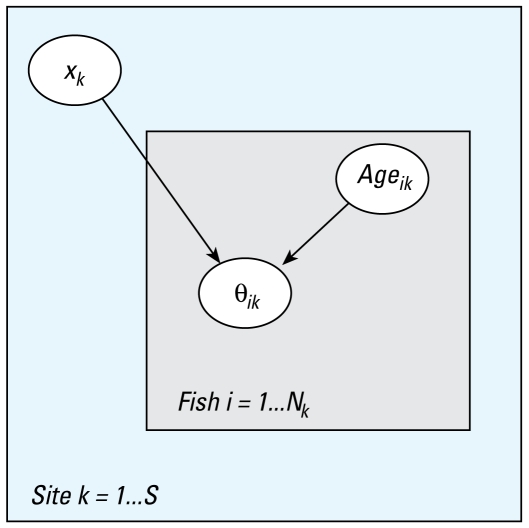
Example of the general form of hierarchical model for a binary response [logit (θ*_ik_*) = β_0_ + β_1_*_k_*age*_ik_* + β_2_x*_k_* + ε*_ik_*, where θ*_ik_* is the probability of response for fish *i* in site *k*, and *x_k_* is the concentration of one of the pollutants at site *k*. Abbreviations: *N*, number of fish; *S*, number of sites..

Because all of the covariates had skewed distributions, they were transformed by *x* → ln(*x* + 1), with 1 added to *x* to avoid difficulties with ln (0) and also so that 0 maps to 0. We used principal components analysis (PCA) to establish the patterns of variation in individual contaminants and hormonal activities in effluent samples collected. We then constructed models describing the relationship between each contaminant (alone and in combination) and each of the biological responses. These were fitted in a step-wise manner, first accounting for the effects due to estrogens and then allowing for additional effects that could be explained by antiandrogens and NP. We used logistic regression to analyze the binary response variables oocytes, fem.duct, and VTG. Generalized linear models (GLM) with gamma-distributed errors (McCullagh and Nelder 1989) fit the response fem.index well. For all responses, the data had a hierarchical structure with varying numbers (12–71) of fish sampled from the 30 sites. The concentrations of each pollutant were at site level, and the response variables were at fish level. A consequence of the data structure was that correlations between fish within sites could be anticipated and needed to be accounted for in the analysis. This was accomplished by first fitting hierarchical GLMs (Gelman and Hill 2007) with random effects for sites. For some responses, variation between sites was not significant; subsequent analyses were then simplified to ordinary nonhierarchical GLMs. An example of the general form of hierarchical model for a binary response is


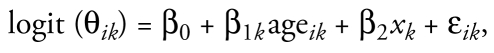


where θ*_ik_* is the probability of response for fish *i* in site *k,* and *x_k_* is the concentration of one of the pollutants at site *k*. This example is represented graphically in Figure 2, in which each rectangle is a level of variation.

Once important covariates were established using these models, we obtained smoothed estimates of the relationships using generalized additive models (GAMs) (Wood 2006). Our aim was to describe the way that covariates interacted with each other in their effect on a response. Surface plots of the fitted models indicate whether pollutants combined in an additive, synergistic, or antagonistic way in their joint effect on the response. Two covariates either *a*) act additively, in the sense that they affect the response independently of each other and the joint effect is the sum of their separate effects; or *b*) interact with each other in their effect on the response. In the latter case, the interaction can be either synergistic or antagonistic.

We performed the statistical computations using R software (R Development Core Team 2007).

## Results

### Exposure predictions

#### *In vitro* hormonal (rYES/rYAS) activity

We predicted that all of the river waters contained estrogenic activity and almost all also contained antiandrogenic activity (Figure 1, Table 1). Predicted estrogenic and antiandrogenic activities in the rivers ranged from 0.04 to 23.21 ng EEQ/L and from 0 to 100.12 μg flutamide equivalents/L, respectively.

#### Concentrations of estrogenic chemicals

After accounting for dilution, predicted steroid concentrations in the rivers receiving the effluents were between 0.01 and 24.09 ng/L for E_1_ and at much lower concentrations for the other two steroids. For some final effluents, we could not identify quantifiable peaks for either the steroids in the effluent extracts or the internal standards in the spiked samples, particularly EE_2_ (present at the lowest concentrations). These samples were noted as no quantifiable peak (NQP). For samples where the analyte was present at a concentration below detection, we assigned a value of one-half the detection limit to the effluent. After adjustment to allow for dilution in the river, these values were near zero. NP and NPnEO were also predicted to be present in river water, with concentrations of NP ranging from 0.003 to 2.079 μg/L. At only 5 of the sites, the concentration of NP was predicted to exceed 1 μg/L in river water.

### Statistical analysis of the distribution of the chemicals

**Figure 3:**
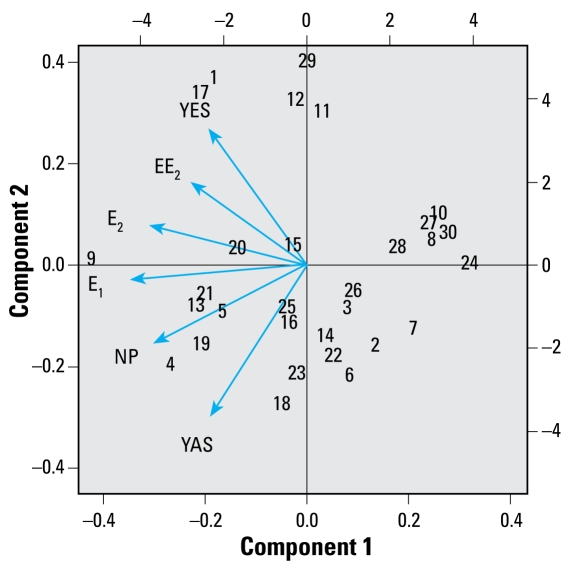
Plot of PCA of the chemical and (anti-)hormone composition of the sample sites showing only the first two components. The numbers on the plot are the site codes listed in Table 1. Component 1 indicates the overall level of contamination. For example site 9 is the dirtiest and site 24 the cleanest. Component 2 is high predominantly for estrogens and low predominantly for antiandrogens. The extremes on this component are sites 18 (antiandrogens) and 29 (estrogens). The arrows represent the variables; two arrows pointing in similar directions indicate that variables are correlated. The top and right axes represent standardized scores for the variables; the bottom and left axes are scores for the sites.

A statistical investigation of the distributions of the various pollutants and hormonal activities present at the sites sampled revealed that many of them were co-occurrent (Table 2). A consequence of the multicolinearity seen in the measurements of the various contaminants was that if the relative proportions of estrogens and antiandrogens were similar across the sites, it would have been difficult to distinguish their separate effects on fish. Fortunately, however, the results of the PCA (Figure 3) revealed that the variation in the chemical composition of the sample sites could be separated into three main components or gradients, including one component (component 2; explaining 24% of the variation in the data) that differentiated the sites with high relative proportions of estrogens from those where antiandrogens predominated. Together, the three components accounted for 87.5% of the variation in the data: Component 1 (50.3%) separated contaminated waters from background, and component 3 (12.4%) was mainly indicative of the concentration of EE_2_ compared with the other steroidal estrogens

### Statistical associations between the chemical exposure and the biological response variables

The results of the PCA analysis indicated that it may be possible to separate the modeling of the associations between the feminizing effects seen in the fish and the anti androgen exposure from those associated with estrogens. The hypothesis that antiandrogens contribute to feminization in wild fish could then be tested using statistical modeling approaches. This was done by first fitting models for each of the biological responses accounted for by estrogens and then estimating any additional effects that could be explained by antiandrogens.

#### Response: oocytes

**Figure 4:**
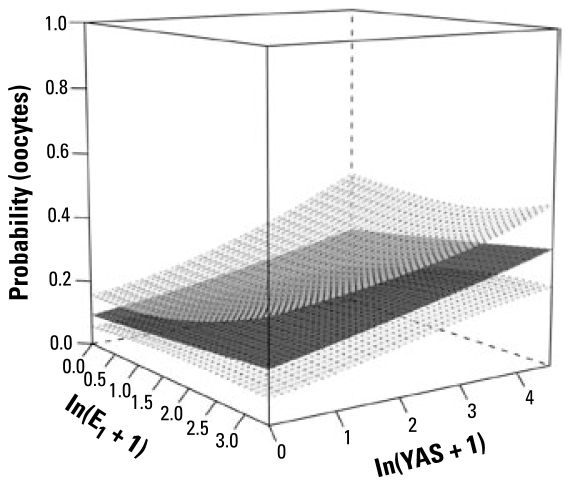
Surface plot illustrating the results of the statistical modeling of the association between E_1_ and anti-YAS and the probability of oocytes in the testes of male fish. The lower and upper surfaces represent 95% confidence limits and the middle surface is the fitted mean. The suggested additive effects were confirmed by the nonsignificant E_1_ × anti-YAS interaction term (*p* = 0.37) in the logistic regression model.

We found 94 cases of fish with oocytes in their testes. The probability of oocytes in the testis of roach was correlated positively with the age of the fish (*p* < 0.0001), with a sharp increase in the age-related effect when the fish were ≥ 3 years of age. Multiple logistic regressions on E_1_, E_2_, and EE_2_, controlling for age, revealed that E_1_ was the most important predictor (*p* = 0.004) of oocytes and that no additional significant variation in the response could be explained by E_2_ or EE_2_ (for EE_2_, there were only 58 cases from sites with reliable estimates of EE_2_ concentration). Because NP was highly correlated with E_1_, it accounted for no additional variation in the response either. Interestingly, we found no correlation between the total estrogenic burden [yeast estrogen screen (YES)] and the *oocytes* response. After allowing for E_1_ and age, however, there was a significant correlation between antiandrogenic activity (anti-YAS) and the *oocytes* response (*p* = 0.01). The surface plot suggested an additive effect of E_1_ and anti-YAS on the probability of oocytes (Figure 4). This was confirmed by the nonsignificant E_1_ × anti-YAS interaction term (*p* = 0.37) in the logistic regression model.

#### Response: fem.index

**Figure 5:**
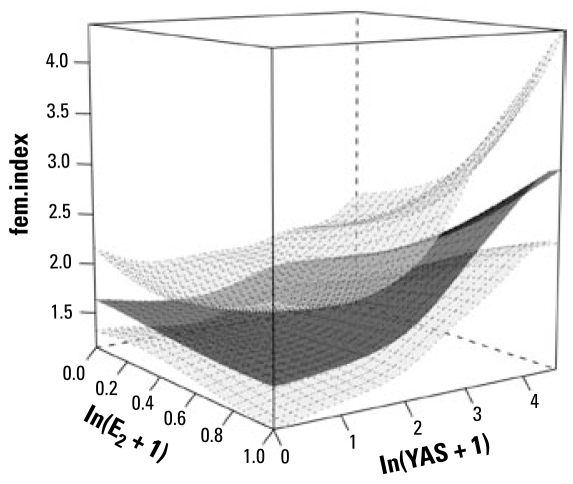
Surface plot illustrating the results of the statistical modeling of the association between exposure to E_2_ and anti-YAS on the feminization index in intersex fish. The lower and upper surfaces represent 95% confidence limits and the middle surface is the fitted mean. The plot indicates a somewhat nonadditive effect of E_2_ and anti-YAS on the fem.index. This was confirmed by a significant negative E_2_ × anti-YAS interaction term (*p* = 0.02) in the logistic regression model.

Of the 94 cases of fish with oocytes in their testes (fem.index > 0), there were only 58 cases for which there were robust measurements of EE_2_ in the WWTW effluents; this was insufficient for use in further statistical analysis. Disregarding EE_2_, multiple logistic regressions on E_1_ and E_2_ revealed that E_2_ was the best predictor of fem.index (*p* = 0.02; averaged over all values of the anti-YAS variable), and there was no effect of NP (*p* = 0.78) or YES (*p* = 0.77) on this response variable. As with the *oocytes* response, after allowing for the effects of E_2_, the additional effect of anti-YAS over E_2_ on the fem.index was significant (*p* = 0.01). The surface plot suggested a somewhat non additive effect of E_2_ and anti-YAS on the fem.index (Figure 5). This was confirmed by a significant negative E_2_ × anti-YAS interaction term (*p* = 0.02) in the logistic regression model.

#### Response: fem.duct

**Figure 6:**
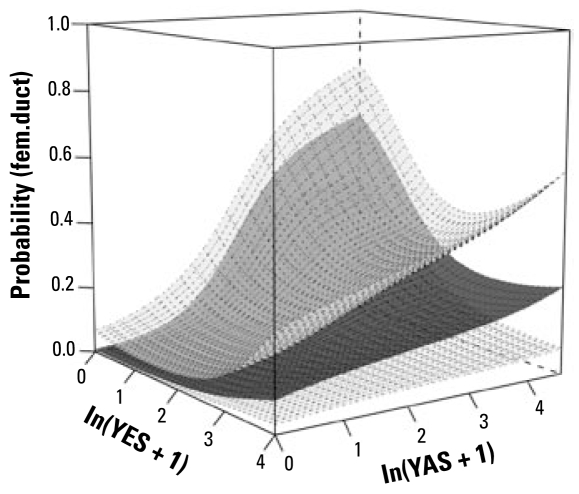
Surface plot illustrating the results of the statistical modeling of the association between exposure to estrogenic and antiandrogenic chemicals. The lower and upper surfaces represent 95% confidence limits and the middle surface is the fitted mean. The plot indicates the additional combined effects of both YES and anti-YAS (*p* = 0.006) over E_1_ on the probability of feminization of the reproductive ducts in wild male fish. The surface plot suggested that there was an increased probability of fem.duct with increased anti-YAS, but that increased YES might partially suppress this response [see Supplemental Material, Figure 6A (available online at http://www.ehponline.org/members/2009/0800197/suppl.pdf) for two-dimensional plot]. This was confirmed by a significant negative YES x anti-YAS interaction term (*p* = 0.01) in the logistic regression model.

We found significant between-site variation (*p* < 0.0001) for the response fem.duct. As explained in “Methods,” we accounted for this intersite variation before testing for covariate effects. Multiple logistic regressions on E_1_, E_2_, and EE_2_ showed that, as with the *oocytes* response, the overall effects of steroidal estrogens on the probability of fem.duct was best explained by E_1_ (*p* < 0.002); again, because NP was highly correlated with E_1_, it accounted for no additional variation in the response. The additional combined effects of both YES and anti-YAS over E_1_ were, however, significant (*p* = 0.006). The surface plot suggested an increased probability of fem.duct with increased anti-YAS, but increased YES might partially suppress this response [Figure 6; see also Supplemental Material (available online at http://www.ehponline.org/members/2009/0800197/suppl.pdf)]. This was confirmed by a significant negative YES × anti-YAS interaction term (*p* = 0.01) in the logistic regression model.

#### Response: VTG

**Figure 7:**
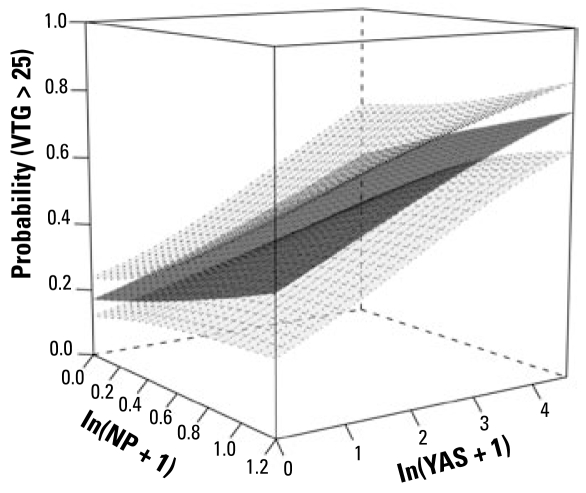
Surface plot illustrating the results of the statistical modeling of the association between exposure to estrogenic and antiandrogenic chemicals on the VTG response in male and intersex fish. The lower and upper surfaces represent 95% confidence limits and the middle surface is the fitted mean. The modeling suggested that NP and anti-YAS were jointly the best predictors of the VTG response, although the contribution of NP was marginal (*p* = 0.09) over the overwhelming effect of anti-YAS alone (*p* = 0.008).

We found significant between-site variation (*p* < 0.0001) in VTG. This was mainly because fish were sampled throughout the year and VTG varies with sampling month. After accounting for this, however, multiple logistic regressions on the steroidal estrogens E_1_, E_2_, and EE_2_ showed that the VTG response was best explained by E_1_ alone (*p* < 0.004). Over and above the steroidal estrogens, NP was a good predictor of the VTG response (*p* = 0.0002). Moreover, there was a very significant effect of anti-YAS on the VTG response (*p* < 0.0001). A comparison of models fitted with all possible subsets of the three variables NP, E_1_, and anti-YAS suggested that NP and anti-YAS were jointly the best predictors of the VTG response, although the contribution of NP was marginal (*p* = 0.09) over the overwhelming effect of anti-YAS on its own (*p* = 0.008). The surface plot suggested that, in general, the VTG response increased with increasing anti-YAS (Figure 7).

When taken together, the results of the statistical analyses suggested that male roach likely exposed to the highest concentrations of antiandrogens and/or steroidal estrogens exhibited the highest prevalence of both ovotestes and oviducts and the highest concentrations of vitellogenin. Moreover, the number of developing oocytes in the testes of the intersex fish (defined by the feminization index) was also the greatest in these fish.

Another important consideration is that, with the exception of the feminization index, the responses seen in the fish did not correlate with the total estrogenic activity present in the water samples as measured by the YES bioassay. Models of the interactions between the total estrogenic activity and the total antiandrogenic activity for each of the responses suggested that estrogenic components of the mixture sometimes appeared to antagonize or reduce responses in the fish that were associated with anti androgen exposure.

## Discussion

These findings support the hypothesis that a combination of steroidal estrogens, nonylphenolic chemicals, and antiandrogens are most likely to cause widespread sexual disruption in wild fish populations in nature. By statistical modeling of the associations between each of the suspected causal factors and the suite of biological effects seen in fish, we established the likely influence of antiandrogens versus estrogens, both alone and in combination, on each response variable. Although these statistical analyses further support the role of steroidal estrogens in the causation of feminization of wild fish in U.K. rivers, they also suggest that antiandrogens are strong causal factors, necessary for severe effects to occur. Indeed, the likely influence of antiandrogenic chemicals on each of the measured responses is clearly demonstrated using a modeling strategy that allows for the effects of steroidal estrogens first before interrogating the data for the existence of additional causal factors. This approach further strengthens the hypothesis that feminization results from the effects of both antiandrogens and estrogens acting in concert.

Sometimes, the antiandrogens appear to act additively with the estrogens to increase a particular response (for oocytes and feminized ducts), whereas in other examples the effect of the antiandrogens appears greater than that of the estrogens (VTG in the blood plasma of males). For fem.duct, we found an interaction between the steroidal estrogens and antiandrogenic activity, the estrogens acting to decrease the response due to the antiandrogens. This does not necessarily imply that all of the factors were interacting to produce a particular response at the same time. Some of the responses (e.g., fem.duct) are induced during early development (e.g., Rodgers-Gray et al. 2001), whereas others (e.g., oocytes) manifest themselves throughout life (Jobling et al. 2006). It is conceivable that when additive relationships are seen, they could be the result of a concentration-related effect of an initiator (acting during early life) and a promoter (acting during adult life).

The estrogenic activity of the water samples (as measured in the YES bioassay) did not correlate well with any of the biological responses or with the concentrations of individual steroidal estrogens measured in the effluents. In most cases, the combined estrogenic activity of the steroidal estrogens present in the effluents was predicted to be higher than that actually measured using the YES bioassay. This lack of correlation between the YES assay results and the individual concentrations of steroidal estrogens could well have been due to the existence of antiestrogenic compounds in some of the effluents, which would reduce the response seen in the YES assay. Indeed the widespread existence of antiestrogenic benzotriazoles in STW effluents, which are potent in the YES bioassay, has recently been reported (Giger et al. 2006). Moreover, Harris et al. (2007) showed that benzotriazoles were not antiestrogenic in fish, even though they were potent anti estrogens in the YES bioassay, thus providing a possible explanation for the mismatch between the fish responses and the YES bio assay response. Indeed, the strong positive correlations of the biological responses with the steroidal estrogen concentrations but not the YES assay results add credence to this suggestion.

Although PCA indicated heterogeneity of antiandrogens and estrogens across sites, there were still correlations between some of the covariates, and the multicolinearity exhibited by these co-occurrent contaminants sometimes confounded the interpretation of the statistical analyses. For example, NP was always highly correlated with E_1_ (Table 2) and so its association with any of the biological effects could rarely be separated from that of E_1_. However, when the strength of the association between one of these parameters and a response was stronger than that of the other, it indicated that the former was a more likely cause than the latter. Intuitively, strong associations are more likely to be causal than weak ones (Hill 1965). Moreover, the statistical modeling strategy we adopted ensured that additional likely causal factors (anti androgenic components) were identified only after accounting for the effects of the main causal factors (steroidal estrogens).

Multicolinearity could also account for the possibility that none of the covariates were causes of feminization in wild fish and that they were masking the identity of an as yet unidentified chemical cause. In most cases, however, this possibility seems highly unlikely, as the association between the antiandrogenic activity and the responses would appear strong enough to rule out hypotheses that the associations are entirely due to one weak unmeasured confounder or other source of modest bias. Moreover, given the fact that laboratory experiments clearly show that exposure to antiandrogens (e.g., Kiparissis et al. 2003; Makynen et al. 2000) or steroidal or xenoestrogens (e.g., Seki et al. 2002; Yokota et al. 2001) can cause sexual disruption in fish, it seems plausible that chemicals with these mechanisms of action could also cause effects in wild fish. For example, inter sexuality and vitellogenin induction can be seen in fish exposed to concentrations of steroidal estrogens in the low nanograms-per-liter range. Moreover, at least with the vitellogenin response, combinations of steroidal (and other) estrogens have been shown to act additively to cause this effect (Brian et al. 2005; Thorpe et al. 2003).

As with estrogenic activity, antiandrogenic activity (given in flutamide equivalents) predicted to be present in the rivers was often sufficient to induce biological responses in fish (Katsiadaki et al. 2006; Kiparissis et al, 2003). In addition, molecular approaches studying changes in gene expression have shown that the feminizing effects of estrogens and antiandrogens in fish share both common and distinct gene pathways (Filby et al. 2007a, 2007b). It seems likely, therefore, that mechanisms exist by which combinations of estrogens and anti androgens could act together when they are administered in combination (Kortenkamp 2008), thus offering further support to some of the cause–effect associations postulated here.

These results clearly demonstrate that induced reproductive health effects in fish in U.K. rivers likely involve factors other than environmental estrogens. The results also provide an interesting parallel with the results of studies performed in rodent models to investigate the suspected environmental causation of testicular dysgenesis syndrome in humans, which is also thought to be mediated primarily by antiandrogenic combined with estrogenic mechanisms rather than by estrogenic mechanisms alone (Christiansen et al. 2008; Sharpe and Skakkebaek 2008; Skakkebaek et al. 2001; Wolf et al. 1999). Although analysis of the human data by itself has so far failed to provide firm evidence of direct causal associations between low-level exposure to specific endocrine-disrupting chemicals and endocrine disorders in humans, studies such as ours that link endocrine effects seen in wildlife to exposure to estrogens and anti androgens present in human domestic waste water may add further credence to the hypothesis that the effects seen in both wild fish and humans are caused by similar combinations of endocrine-disrupting chemical cocktails to which both fish and humans are exposed.

## Figures and Tables

**Table 1:** Exposure predictions and biological impacts for 30 river sites around the United Kingdom.

Site	E_2_ (ng/L)	E_1_ (ng/L)	EE_2_ (ng/L)	YES EEQ (ng/L)	Anti-YAS flutamide Eq (μg/L)	NP (μg/L)	Ovotestes (*n*)	Oviducts (*n*)	Mean		
									VTG male	VTG intersex	Intersex index
1	NQP	0.42	NQP	0.14	9.39	0.15	0	1	—	25	—
2	0.3	5.2	< 0.25^a^	2.1	51.7	1.05	3	0	25	32	2.28
3	0.366	9.42	0.203	0.37	12.77	0.386	2	6	—	39	1.25
4	< 0.066	5.69	< 0.039	23.21	0	0.345	1	0	188	NS	1.33
5	< 0.021	0.1	< 0.012	1.24	0	0.09	5	0	496	2,332	1.54
6	< 0.25	< 1	< 0.15	1.95	0	0.2					
7	1.308	3	0.099	1.63	6.18	0.318	4	3	310	305	1.42
8	0.115	2.13	< 0.043	0.75	29.29	0.542	7	2	273	793	1.90
9	NQP	1.26	NQP	0.31	11.31	0.344	3	1	84	525	1.79
10	NQP	14.72	NQP	4.77	70.63	1.353	5	9	202	125	1.44
11	0.198	1.26	0.331	0.79	50.41	0.553	1	2	142	25	1.17
12	< 0.08	5.03	< 0.05	7.96	0	0.851	5	1	42	25	1.70
13	< 0.005	0.01	< 0.003	0.04	0	0.003	0	0	16	—	—
14	0.881	4.56	0.116	1.71	5.77	0.247	6	6	34	43	1.67
15	0.991	4.96	0.159	1.07	24.26	0.557	6	3	477	487	2.05
16	NQP	15.95	NQP	45.1	0	0.70	2	3	81	10,617	1.17
17	NQP	2.53	NQP	0.67	0	0.072	2	0	37	75	2.52
18	NQP	0.95	NQP	2.94	5.65	0.053	1	0	22	10	1.33
19	0.548	2.06	0.058	1.18	13.30	0.251	3	3	25	51.8	3.28
20	< 0.179	3.1	< 0.108	0.79	100.12	0.618	3	8	69	334	1.5
21	< 0.152	15.23	< 0.091	1.1	19.55	0.82	7	11	7,022	20,907	2.36
22	2.799	24.09	< 0.106	7.09	72.21	1.303	7	1	41	186	3.43
23	< 0.0013	0.44	< 0.0008	0.85	0	0.023	0	0	25	—	—
24	1.086	9.84	0.1	3.94	75.14	0.796	4	6	422	272	2.17
25	< 0.092	0.24	< 0.0923	1.1	0	0.094	1	0	25	27	1.17
26	< 0.052	3.42	< 0.031	0.12	10.93	0.739	0	3	25	246	—
27	< 0.063	3.56	< 0.038	0.33	22.74	1.723	1	8	208	426	1.33
28	0.23	1.6	0.177	0.34	9.436	0.255	6	5	25	37	1.77
29	NQP	18.16	NQP	1.15	17	2.079	8	13	179	203	1.52
30	< 0.25	2.0	< 0.15	5.1	0	0.7	0	0	122	—	—

Abbreviations: EEQ, estradiol equivalents; flutamide Eq, flutamide equivalents; NQP, no quantifiable peak (no data); NS, not significant. Concentrations of E_2_, E_1_, EE_2_, and NP, as well as total estrogenic activity (EEQ) and total antiandrogenic activity (flutamide Eq) were predicted (from effluent concentrations and dilution factors).

a The “less than” symbol (<) indicates effluent samples in which the concentration of the desired analyte was below the detection limit; the detection limit in each case was divided by the dilution factor of the effluent in the river at the point where the fish were captured.

**Table 2:** Statistical investigation [correlation coefficients (*r*)] of the co-occurrence of the various pollutants and hormonal activities present in the effluents sampled.

	E_1_	E_2_	EE_2_	NP	YAS
E_2_	0.72#				
	*n* = 28				
EE_2_	0.35*	0.56**			
	*n* = 22	*n* = 22			
NP	0.77#	0.45*	0.26 NS		
	*n* = 30	*n* = 28	*n* = 22		
YAS	0.48**	0.22 NS	0.00 NS	0.62**	
	*n* = 30	*n* = 28	*n* = 22	*n* = 30	
YES	0.49**	0.44*	0.51*	0.15 NS	−0.25 NS
	*n* = 30	*n* = 28	*n* = 22	*n* = 30	*n* = 30

NS, not significant. The steroidal estrogen E_2_ and its metabolite E_1_ were highly correlated (E_2_ is oxidized to E_1_). EE_2_ (the contraceptive pill hormone) was also associated with E_2_, as expected. We found no correlation between the total estrogenic (YES) and total antiandrogenic (anti-YAS) activities, indicating that the chemicals inducing these two hormonal activities are likely to be different.

* *p* < 0.05.

** *p* < 0.01.

# *p* < 0.001.
